# Analysis iron distribution methods in fine sand- and silt-sized soil particles

**DOI:** 10.1016/j.mex.2021.101248

**Published:** 2021-01-29

**Authors:** Tong He, Juan Li, Jessica Gray, Ying Gu

**Affiliations:** a School of Geographic Sciences, Nanjing University of Information Science and Technology, China; b State Key Laboratory of Mineral Deposits Research, School of Earth Sciences and Engineering, Nanjing University, China; c Julius Kruttschnitt Mineral Research Center, Queensland University, Australia

**Keywords:** Lithogenic particles, Iron partition in silicate phases, Single-particle mapping technique, Iron provenance

## Abstract

Iron is prone to change its form and speciation in phases. Thus, several methods have been developed to estimate iron partitioning in the mineral phases of soils. However, the accuracy of these methods to evaluate the iron contribution from minor phases, such as actinolite, almandine, biotite, chlorite, epidote, hornblende, muscovite, and Fe-diospide, remains low. Furthermore, most of the current iron speciation research is focused on bulk samples, and only applies to soil samples that are mostly composed of clays or clay minerals, without a wide-ranging evaluation of soil particles with different grain sizes. In this study, we classified several iron phases using a mineral liberation analyzer on desert soil particles with diameters ranging from silt- to fine-sand (5–20 µm, 20–45 µm, 45–63 µm, 63–75 µm, and > 75 µm). The iron containing minor phases were identified, the modal mineral abundances were determined via matching with the standard energy dispersive spectra library, and a particle size analysis was performed using mineral processing tools on each of the examined 40,000 particles. The iron partition results were ultimately established based on the standard iron concentration in the mineral phases and the modal mineral abundances.

• This new method could be automated, thereby facilitating high efficiency identification of iron-containing phases that would allow, for the first time, the generation of a dataset for iron partitioning in soil particles.
• This method allows the identification of minor iron phases in soil particles, and permits in situ mapping of iron mineralogy in fine sand- to silt-sized soil particles.
• Not restricted by single mineral particles, this method considers multi-phase complex particles. Thus, it largely improves the accuracy for estimating the iron partition parameter.

## Graphical abstract

**Figure:**
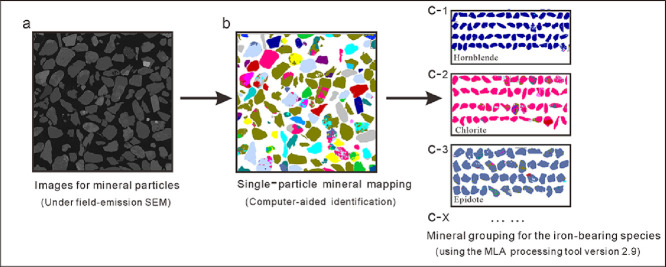

Specifications table

**Table:** 

Subject Area:	Earth and Planetary Sciences
More specific subject area:	Geochemistry, paleoclimate, paleogeography
Method name:	Iron partition in soil particles based on in situ single-particle mineral mapping technique
Name and reference of original method:	Fandrich, R., Gu, Y., Burrows, D., Moeller, K. Modern SEM-based mineral liberation analysis, International Journal of Mineral Processing. 84 (2007) 310–320. doi.org/10.1016/j.minpro.2006.07.018
Resource availability:	*If applicable, include links to resources necessary to reproduce the method (*e.g.*, data, software, hardware, reagent)*

## Background

The automated mineralogy technique has been widely applied in provenance studies [1], [2], [3], [4]. A variability estimation recommends that modal mineral analysis using mineral liberation analysis (MLA) is the preferred approach for reporting mineral concentrations [2]. While a multi-sample comparison of the modal mineral abundances could be performed directly, it is not convenient when the number of analyzed single particles is small [5] as a small sample size may introduce uncertainties in the dissimilarity estimates [6]. Therefore, to achieve better statistical validity, the target number of single particles to be analyzed for each sample was determined to be 40,000.

## Experimental design and parameter settings

We selected silt-sized (20–75 µm) and fine silt-sized (5–20 µm) fractions to study silicate mineral concentrations using an MLA. A previous study revealed that an abundance of clay-aggregates dominated in each fraction size in the soils [7]. Considering the dominant species is clay aggregates, the mineral compositions would vary little throughout the different size fractions and distinct sampling sites. Thus, during pretreatment, we removed the clay aggregates. The desert sand samples were pretreated by wet sieving and ultrasonic cleaning to remove most of the clay-aggregate particles, even though clay aggregates are not likely to be an important species in desert sand dune sediments. However, if any clay-cemented aggregates survived ultrasonic cleaning, they generally displayed complex X-ray spectrum that did not match any known mineral phase spectra in the standard mineral library.

An MLA-650 system is composed of a Quanta 650 SEM and dual EDAX energy dispersive spectral analysis (EDS) detectors, wherein the frame resolutions were set to 1024 × 800. Image brightness and contrast were calibrated using a gold pin in the standard block on the stage. The gray level of the gold pin for each backscattered electron (BSE) image was set to approximately 230, while the background (resin) setting after carbon coating was approximately 20. A low noise, high-resolution image for each single particle was obtained. The detected single particles revealed a higher gray level than the epoxy resins. The BSE image for each particle was analyzed to obtain its geometric parameters, including length, width, and particle boundary area [8]. These high-resolution (0.1 µm) images provide information that could accurately discriminate the mineral phases within each single-particle.

## Novelty of this methodology

An EDS spectrum pattern enables us to identify the mineralogical makeup of a single particle in dust [9] as it quantifies the chemical compositions of each single particle [10]. Then, the obtained EDS spectra are automatically compared with the standard pattern in the mineral library. A high threshold value is set to discriminate the similarities between the patterns [8] to ensure that the minerals are correctly identified. If the EDS spectrum is different from any of the patterns in the library, the single particle with this spectrum is automatically grouped into the “unknown”. Each single-particle in the “unknown” is individually checked and the EDS spectra are recollected using the “MLA processing tool”. In general, the “unknown” particles have complex X-ray spectra that are identified as clay-aggregates [9]. The clay-aggregate single particles are not considered in the final template of the modal mineral abundance.

**Fig. 1:**
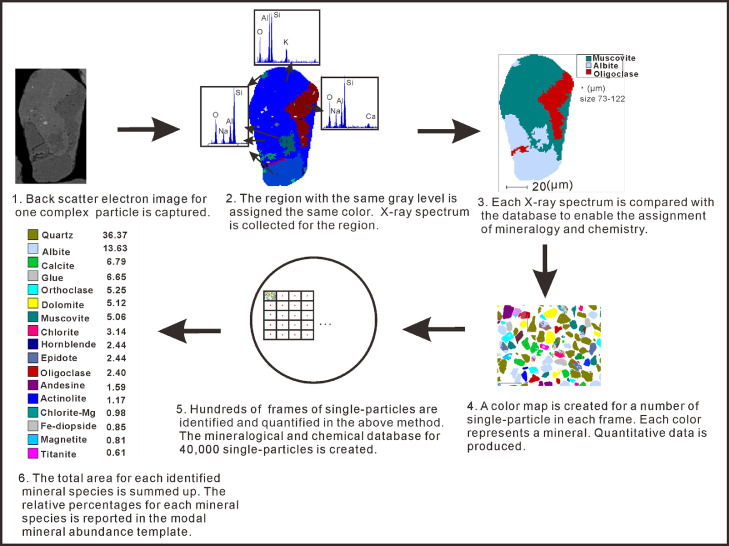
Schematic diagram outline of mineral liberation analysis (MLA) modal mineral abundance measurement method that systematically measures single-particles.

Regarding complex particles (e.g., three mineral phases in one particle), the traditional point-counting method used in the SEM-EDS system [9] is not often capable of identifying the mixed mineralogy and complicated phases within a particle for a large number of single particles. Thus, a digital image of the single particle is constructed pixel by pixel using thousands of X-ray measurements before it undergoes processing using the “MLA processing tool” (Fig. 1). This process is known as single particle mapping [8]. Phase segmentation for an enlarged BSE image for one complex single particle is created by assigning a unique color for regions of homogeneous gray levels (Fig. 1). A gray level may coincide with that of one particular mineral phase in the same particle as a result of the same average atomic numbers. Once the complex single-particles are identified, the next step of the analysis focuses on the identification of simple phase single-particles. Thus, the phase area in either the simple-phase particle or the complicated particle are determined. This method produces area-based percentages of the sample's mineral components (Fig. 1). These area percentages are converted to weight percentages using mineral density, and these values are reported as the modal mineral abundances.

## Method validation

The chemical concentration, specifically the iron concentration, in each of the silicate mineral phases is obtained from the EMPA measured data [11]. Note that the data template is listed in Supplementary Table 1S. The iron concentration of each silicate mineral phase is multiplied by its modal abundance (Supplementary Table 2S), which is determined by Eq. (1). The total iron content in all the identified silicate phases containing iron is calculated using Eq. (2). As a percentage of the total silicate-bearing iron content, the iron content in one specific silicate mineral was used as its iron partition (%), which is expressed by Eq. (3). Here, we use the relative iron partition (%) for each of the identified silicate phases containing iron as the best approximate estimation of iron speciation (Supplementary Table 2S). Thus, the quantitative iron speciation is determined using Eq. (3). For greater simplification, the parameters in these equations use abbreviations, wherein k_i_ is the iron concentration in mineral phase i, C_i_ is the weight percentage of mineral phase i, F_i_ is the weight percentage of the iron content in mineral phase i in the total mass, F_sili_ is the total iron content in all identified silicate phases containing iron, and P_i_ is the relative percentage of iron content in mineral phase i versus the total iron content in all identified silicate phases containing iron.

(1) Fi=ki*Ci,

(2) $$F_{\text{sili}}=\sum_{i=1}^{n}\;k_{i}\;*C_{i}\;,$$

(3) $$\text{Pi}\;=\;\text{Fi}\;/\;F_{\text{sili}\;}*\;100\%,$$

## Declaration of Competing Interest

None.

## Appendix Supplementary materials

Image, application 1

Supplementary material *and/or* Additional information Appendix Supplementary materials Table 1S and Table 2S. Image, application 2
